# What Do You Know about Twilight Sleep?

**Published:** 1914-09

**Authors:** F. E. Daniel


### EDITORIAL DEPARTMENT.

MRS. F. E. DANIEL, Publisher and Managing Editor.
DR. R. H. L. BIBB, Associate Editor.

CONTRIBUTING EDITORS:

Dr. H. Sheridan Baketel, New York, editor *Medical Times:* Dr. M. H. Boerner, Austin, Texas; Dr. G. Henri Bogart, Paris, Ill.; Dr. M. M. Carrick, Dallas, Texas; Dr. Edward H. Carey, Dean Medical Department, Baylor University, Dallas, Texas; Dr. W. L. Crosthwaite, Waco, Texas; Dr. T. D. Crothers, Hartford, Conn.; Dr. H. W. Cummings, Hearne, Texas; Dr. Oscar Dowling, New Orleans, President Louisiana State Board of Health; Havelock Ellis, M. D., London, Eng.; Dr. May Agnes Hopkins, Dallas, Texas; Dr. A. W. Fly, Galveston, Texas; Dr. G. B. Foscue, Waco, Texas; Dr. E. S. Goodhue, Holualoa, Kona, Hawaii; Dr. M. B. Grace, Seguin, Texas; Dr. Joe Gilbert, Austin, Texas; Dr. Charles W. Hughes, St. Louis, editor *Alienist and Neurologist*; Dr. James G. Kiernan, Chicago; Dr. George H. Lee, Galveston, Texas; Dr. John T. Moore, Houston, Texas; Dr. Frank Paschai, San Antonio, Texas; Dr. John Preston, Austin, Texas, Superintendent State Lunatic Asylum; Dr. Bacon Saunders, Fort Worth, Texas; Dr. Allen J. Smith, Philadelphia, Medical Department, University of Pennsylvania; Dr. J. T. Scott, Austin, Texas; Dr. Ralph Steiner, Austin, Texas, President Texas State Board of Health; Dr. John S. Turner, Dallas, Texas; Dr. Charles Venable, San Antonio, Texas.

### What Do You Know About Twilight Sleep?

In the August number of the *Ladies' World*—one of the McClure publications—appeared an article entitled "The New Birth or the Twilight Sleep." *The Lancet-Clinic* declares:

"The article is anonymous except that Professor Krönig and Dr. Gauss are quoted extensively, and one American woman writes a letter very much like those we are familiar with in the quack advertisements of our daily press, except that it is very much better done—so much better done, in fact, that we might imagine that Marguerite Tracy or Constance Leupp had done it."

In the article was reproduced the pictures of Dr. Krönig and Dr. Gauss, but Dr. Krönig declared that McClure's had bribed some underling of the *Frieburg Clinic* to furnish this material.

The clever editor of the *Lancet-Clinic* is rather cynical about Dr. Krönig's knowledge of this matter, and thinks that his feeble denial of his complicity in the publication of the articles justified the McClure's in carrying out what he terms:

"The most gigantic advertising venture that the world has yet seen—namely, the placing of the *Ladies' World* in the hands of every 'thoughtful American family' 'within one year.' "

In order to facilitate the subscription list, the McClure's are offering a popular treatise on obstetrics absolutely free—to those who send $1.00 for a year's subscription to the *Ladies' World.* This book is entitled "Painless Childbirth," and is from the pen of Henry Smith Williams.

To a casual observer it would seem, however, that the book is an advertisement for the Frieburg Hospital, as will be seen by some of the titles of chapters, viz.: The Need of a Painless Method; Percentage of "Assisted Births" Alarming; the Dangers of Chloroform; First Experiments at Frieburg; The Great Obstetrical Authorities; Scopolamine—Its Uses, Early Discouragements, Success; The First American Baby; Dr. Krönig's Opinion; Six Typical Cases, Names and Addresses.

It appears that the *McClure's* feel that the doctors of the country are not doing their full duty, and that they must educate and enlighten the public, for they state that "the purpose of this book is to supplement the work now being done by the *McClure* group of magazines in spreading this *new gospel of hope for women.*" And to further convince the public that the doctors are not doing their duty they state that it was they who first introduced salvarsan in the United States and that they uncovered the dangers of ankylostomiasis.

In giving their reason for publishing these articles, they say:

"These articles are published to create a public demand for painless childbirth in this country. The American women, who have to bear the children, must rouse the interest, sympathy and faith of the American medical profession—the most skillful, but the most ultra-conservative body of men in the world. Naturally, the average physician is not primarily interested in abolishing pain in childbirth, especially when it means a long period of study and experiment and expense. The average woman is interested, vitally interested—and what she wants, she gets. Two points should be emphasized: First, that 'The Twilight Sleep' should not, and cannot safely, be practiced in this country without the co-operation of the leading physicians. Second, that every effort should be made to secure that co-operation; if necessary to force it."

So the *McClure* people actually believe that "the average physician is not primarily interested in abolishing pain in childbirth, especially when it means a long *period of study* and *experiment* and *expense.*" Can it be possible that men who are supposed to be so well informed on any and all subjects know so little about the medical profession as to make a statement of this kind? Does the publisher hope his "sop to Ceberus," in the statement that the "American medical profession is the most skillful, but the most ultra-conservative body of men in the world," will for one moment prove as oil to the slanderous wound made in the statement that "the average physician is more interested in the *experiment* and the *expense*" than he is in relieving pain?

Maybe we should not take these publishers so seriously, for the very next utterance proves them very fallible indeed, for they state that the "Twilight Sleep should not and cannot be safely practiced in this country without the co-operation of the leading physicians." Well let us see what some of our leading physicians have to say in regard to this matter.

In the *Ladies' Home Journal* for September appears letters from four of the most eminent obstetricians in the United States.

Dr. Charles Green, Professor of Obstetrics in Harvard, says that, in 1903, he favored this therapeutic method of producing "Twilight Sleep," and wrote an article to that effect, but that he has long since abandoned it for two reasons:

"First, because it has apparently been the cause, occasionally, of fetal asphyxia.

"Second, because the effect of the drug on the mother is often uncertain, and unless used with great care may cause unfavorable and dangerous results."

Then he adds:

"Moreover, we have other and safer measures for the relief of pain in labor. So I have given up teaching the use of scopolamine in my lectures."

Dr. J. Whitridge Williams, Professor of Obstetrics in Johns Hopkins, has this to say regarding the treatment:

"We have used the scopolamine treatment of childbirth in two separate series of cases at the Johns Hopkins Hospital. But in neither series were the results satisfactory, nor did they in any way approach the claims made for the treatment. We expect to do more with it next year. In the meantime my own experience and conversation with Professor Krönig do not make me feel that the method really constitutes a great advance over those which are in use by American physicians."

Dr. Barton Cooke, Professor of Obstetrics in the University of Pennsylvania, says that as early as "1903 American physicians tried this method in some of our largest maternities." In the Maternity of the University of Pennsylvania it was tried in a series of cases which extended over a period of two years. After having visited Frieburg, and observed this method in clinic, Dr. Cooke is of the opinion that the results obtained were partly psychological, as the patients were told beforehand that there would be no suffering, and were told after delivery that they had had no pain.

Dr. Joseph B. De Lee says that in 1913 he spent four weeks in the Frieburg clinic, witnessing in all ten cases of childbirth, and that the impressions he received were "decidedly unfavorable," and he gives the following reasons:

"In all the ten cases the birth pains were weakened and labor prolonged; in two of the women for almost two days. In three cases pituitrin had to be given to save the child from imminent asphyxia.

"In five of the cases instruments had to be used. In my opinion two of these were directly rendered necessary by the paralyzing effects of the drugs scopolamine and morphine. Extensive lacerations resulted.

"Several of the women became delirious and so unruly that ether had to be administered in addition to the scopolamine and morphine, the result being that the infants were born narcotized and asphyxiated to a degree. One had convulsions for several days.

"All these occurrences confirmed my own experience with the drugs. I had used them when first proposed twelve years ago. At that time they were extensively employed in Europe and America, but were soon discontinued because they were found impractical and dangerous."

And not only did the doctor visit Frieburg; he continued his investigation, to find that in Berlin, Vienna, Munich and Heidelberg the scopolamine method had been tried and discarded.

The letters prove conclusively that the statements made by *McClure's* are inaccurate and misleading, as the American physicians have tried "scopolamine method" years and years ago.

The *Woman's Home Companion* for September devotes more than a page to "The Twilight Sleep at Frieburg." The article is written by William Armstrong, who is not a physician, but it was submitted by the editors to Dr. Ross McPherson, of New York, who says very candidly that he has used this *method extensively* and he believes it to be "decidedly overestimated."

But in spite of this statement the editors of the *Ladies' Home Companion* insists: "The Twilight Sleep is not yet available in America, and will not be for a considerable period."

Will someone please explain this statement for us. We fail to grasp the significance. If it has been in use for years, why is it "not yet available?"

The editor of *Clinical Medicine* in the July issue says, in speaking of the administering of these "made in Germany" drugs:

"The procedure is already well known to thousands of physicians in the United States, has been unostentatiously practiced by them for at least a decade, and long ago passed out of the experimental stage into the established routine of the lying-in room."

We heartily agree with him when he expresses himself as:

"Almost tempted to suspect that such an article as the one referred to crept into the reading pages by mistake (or otherwise) and properly belongs in the paid advertising space. At any rate, the net effect of such conspicuous publication is to blow the horn of the Freiburg clinic and to create the misleading impression that it is necessary to go thence in order to enjoy the advantages of the so-called 'twilight sleep.'"

We do not understand, though, why he should offer an apology to Gauss and Krönig for what he had written, for we feel sure if an American physician had perpetrated such an extensive advertising scheme of his hospital and himself he would have been ostracised and branded forevermore as the "king of quacks." But, of course, we are not meaning to intimate that our friend, the editor, is influenced by any foreign brand of men or medicine.

In the August issue of this same journal appears a letter from a woman who styles herself as "A Humane Woman," but the way she goes after the doctors of this country does not seem very "humane," and somehow her letter savors very strongly of McClure-ism. But the editor published it and then proceeded to take the medical profession to task for being so slow to accept innovations, and he places the blame where it justly belongs, viz., not with the profession at large, but on "constituted medical authority."

*McClure* certainly does owe the medical profession of the United States an apology for his unwarranted and unjust attack upon them, for we find upon investigation that even in poor benighted, crude, semi-barbarous Texas the physicians long ago tried this "scopolamine method" and discarded it because the results were so unsatisfactory and so uncertain. So we must conclude that there are some few doctors yet alive who do hold human life dearer than "long periods of study and *experiment* and *expense.*"

Perhaps the doctor of America can find some comfort in the words of Marcus Aurelius, who wisely declared: "If a man standing by a lovely spring should rail at it, the water is none the worse for his foul language; and if he threw in dirt it will quickly disappear, and the fountain will be as wholesome as ever."

MRS. F. E. DANIEL.

